# Electron Beam-Degraded β-Glucans from *P. ostreatus* Retain Their Structure and Biological Activity

**DOI:** 10.3390/polym18111363

**Published:** 2026-05-30

**Authors:** Zhanna Lyutova, Alexandra Dozortseva, Yakov Manurikov, Maria Markova, Anton Mazur, Vladislav Khaleev, Alexandr Arutyunyan, Alina Borisenkova

**Affiliations:** 1 Radiation Technology Department, St. Petersburg State Institute of Technology, Technical University, 190013 St. Petersburg, Russia; 2 Petersburg Nuclear Physics Institute Named by B.P. Konstantinov of National Research Centre “Kurchatov Institute”, 188300 Gatchina, Russia; 3 Microbiological Synthesis Technology Department, St. Petersburg State Institute of Technology, Technical University, 190013 St. Petersburg, Russia; 4 Chemical Technology Institute, Yaroslav-the-Wise Novgorod State University, 173003 Veliky Novgorod, Russia; 5 Research Resource Center “Magnetic Resonance Research Methods”, St. Petersburg State University, 199034 St. Petersburg, Russia

**Keywords:** water-soluble β-(1→3,1→6)-glucans, *Pleurotus ostreatus*, E-beam irradiation, *Apis mellifera carnica*, honey, proline, feeding, honey bee

## Abstract

β-glucans are biologically active biopolymers that influence metabolic activity and possess immunomodulatory, antitumor, anti-inflammatory, and wound-healing properties. The biological activity of β-glucans is influenced by both their primary structure and spatial conformation, which, in turn, depend on the natural source of the polysaccharides and their extraction methods. In this study, the water-soluble fraction of β-(1→3,1→6)-D-glucan, isolated from non-irradiated and irradiated *Pleurotus ostreatus* cryopowder pre-treated with alcohol, was investigated. Increasing the irradiation dose was found to increase the yield of the water-soluble glucan fraction. Furthermore, irradiation increased the solubility of glucans in water and reduced their bulk density and moisture content, which may be due to partial degradation of the biopolymer. According to FTIR and NMR spectroscopy, water-soluble glucan samples isolated from both irradiated and non-irradiated samples consist predominantly of β-(1→3,1→6)-D-glucan, protein, and a small amount of (1→3)-α-glucan. It was found that the triple-helix conformation of glucans in solution, assessed using Congo red analysis, did not change upon irradiation. Irradiation resulted in a dose-dependent increase in the antiradical activity of the glucans against the stable DPPH radical. Addition of irradiated glucans to sugar syrup used as a feed supplement for bee feeding resulted in a significant increase in the proline content of honey. Thus, electron irradiation is a promising method for obtaining β-glucans with high water solubility and contributes to enhancing their biological activity.

## 1. Introduction

β-glucans are a class of glucose homopolymers linked by β-1→3, β-1→4, or β-1→6 glycosidic bonds, or combinations thereof, that are found in the cell walls of a wide variety of natural sources, including cereals, bacteria, fungi, and grains. β-glucans can modulate host immune responses by priming and stimulating innate immune cells such as macrophages, neutrophils, and granulocytes. Both in vitro and in vivo studies have shown that both soluble and insoluble β-glucans possess immunostimulatory activity through different pathways [1,2]. Thus, insoluble β-glucans more effectively activate Dectin-1-dependent pathways, while soluble ones mediate immunomodulation by activating CR3-dependent pathways and the complement system [2]. Furthermore, structural differences in β-glucans obtained from different sources induce variable immune and antitumor responses [3]. It should be noted that a clear structure-functional relationship determining the pharmacological properties of β-glucans has not yet been established. Some studies indicate that a maximum cellular response is possible only when the cell binds to β-glucans with a high molecular weight (400–2000 kDa) and a complex tertiary structure, as in this case, a large number of receptors are simultaneously involved in the reaction [4,5,6]. The antitumor activity of β-glucans is also largely attributed to its helical conformation [5,7]. On the other hand, some studies have shown that immunostimulatory activity was higher in lower molecular weight polysaccharides [8,9]. Also, some studies have shown that β-glucans with a single-chain structure had higher biological activity than polysaccharides with a triple helical structure [8,10]. Therefore, the relationship of molecular weight and conformation of β-glucans with biological activity still needs further study.

One of the effective ways to reduce the molecular weight of β-glucans, and, as a consequence, increase their solubility, is their radiation modification [11]. Moreover, it was shown that radiation-treated low molecular weight glucans had increased immunostimulating [12], antioxidant [13,14], antibacterial [15] and antihyperlipidemic activity [16,17], and antitumor effect in vivo and in vitro [18]. Thus, it was found that the activity of irradiated β-glucan in stimulating RAW 264.7 macrophage cells was higher than that of unirradiated β-glucan [19]. Furthermore, oral administration of gamma-irradiated β-glucan significantly increased proliferation and cytokine (IFN-γ and IL-2) release by spleen and Peyer’s patch cells compared with unirradiated β-glucan. Degraded β-glucan with a molecular weight of about 25 kDa, obtained by γ-irradiation, is a very promising ingredient for use in nutraceutical products for the treatment of diabetes and dyslipidemia [20]. Radiation-treated β-glucan has also shown great promise as a feed additive to improve the survival of broilers [21,22], whiteleg shrimp *Litopenaeus vannamei* [23], and honeybees [24,25].

Thus, we hypothesize that radiation treatment can significantly alter the biological activity of β-glucans. In our study, we used the proline content of polyfloral honey produced by bees fed β-glucans as a dietary supplement as a parameter to assess the impact of radiation modification on the biological activity of β-glucans. The proline in honey, on the one hand, is an important biomarker for assessing the nutritional status of honey bees, the efficiency of nectar collection and the viability of the colony [26,27,28], and on the other hand, its content is a recognized, standardized indicator of honey authenticity and purity [29].

The aim of this study was to determine the effect of accelerated electron irradiation on the structure, physicochemical properties, and antiradical activity of water-soluble β-glucan extracted from *P. ostreatus* biomass, as well as to determine the effect of the resulting products on the proline content of honey when used as a food supplement for *Apis mellifera carnica* bees.

## 2. Materials and Methods

### 2.1. Extraction of Water-Soluble Glucans from P. Ostreatus Biomass

In this work, the source of glucans was cryopowder (Production Association GAMMI JSC, Nizhny Novgorod, Russia) obtained from the biomass of the fungus *Pleurotus ostreatus*. The first stage of glucan isolation from the cryopowder involved performing an alcohol extraction to remove lipophilic compounds and low molecular weight components. A sample of the cryopowder (40.0 g) was placed in a round-bottom flask, mixed with 80% ethanol at a mass ratio of 1:9, and heated on a heating mantle. The extraction was carried out for 3 h from the moment boiling began. The precipitate was then filtered off, and the process was repeated. After two alcohol extractions, the biomass was filtered, dried at a temperature of 60–65 °C, and ground. At this stage, an insoluble glucan-containing preparation (32.0 ± 0.5 g) was obtained. The insoluble powder was irradiated with doses of 25–75 kGy (dose rate of 0.1 kGy/s) at room temperature. Irradiation was performed with the high-energy electrons accelerated to 10 MeV on a Mevex MB10-30SC900 linear accelerator (STERIS AST Equipment and Technologies, Mentor, OH, USA). Dose control was carried out using a film dosimeter CO(PDE) 1–10 (VNIIFTRI, Zelenograd, Russia). To obtain a soluble glucan-containing preparation, the second isolation stage—water extraction—was carried out. As a control experiment, extraction of non-irradiated insoluble preparation was also carried out. The water extraction began by boiling the insoluble irradiated preparation in distilled water at a preparation-to-water ratio of 1:75 for 3 h. The water level was replenished to the original volume throughout the boiling process. After 3 h, the precipitate was filtered off, re-suspended in 3 L of water, and the procedure was repeated once more. Upon completion of filtration after the second water extraction, the filtrates from both procedures were combined and concentrated on a rotary evaporator to a volume of 50–100 mL. Subsequently, glucans were precipitated from the obtained concentrated filtrate (cooled to room temperature) using 96% ethanol at a ratio of 1:5. To ensure complete precipitation, the vessel containing the precipitated glucans was placed in a refrigerator for 10–12 h. Then the precipitate was filtered, dried at a temperature of 60–65 °C, and ground. All experiments were performed in triplicate. Table 1 presents the weights of the final water-soluble glucan-containing products.

The yield of the preparation was calculated as the ratio of the weight of water-soluble glucan obtained from irradiated or non-irradiated insoluble glucan to the weight of the insoluble drug obtained in the first stage of glucan extraction.

### 2.2. Characterization

IR-Fourier spectroscopy was performed on an IRTracer-100 spectrometer (Shimadzu, Kyoto, Japan). The spectra were recorded at 32 scans per spectrum and a resolution of 4 cm^−1^ in the range of 4000–400 cm^−1^. The analyzed substances dispersed with potassium bromide were pressed into pellets. The absorption spectra were obtained using UV-Vis spectroscopy on an SF-2000 spectrophotometer (OKB Spectr LLC, St. Petersburg, Russia). The solid-state ^13^C NMR spectra were obtained on a Bruker Avance III 400WB spectrometer (Bruker Corporation, Billerica, MA, USA). A two-channel sensor equipped with a magic angle sample rotation (MAS) system was used. The sample was rotated at a frequency of 10 kHz in a 4 mm zirconium oxide rotor. For excitation, a cross-polarization sequence (CP/MAS) was used with a 2 s relaxation delay time and 2 ms contact times. A sequence of direct excitation with decoupling from protons with a 30 s relaxation delay time and 3.2 µs of exciting pulse duration. The ^1^H NMR spectroscopy was carried out on a Bruker BioSpin AG Avance III HD 400 spectrometer (Bruker Corporation, Billerica, MA, USA). Tetramethylsilane (TMS, Sigma Aldrich, St. Louis, MO, USA) was used as an external standard.

### 2.3. Moisture Content Determination

The moisture content of native and irradiated glucan-containing samples was determined gravimetrically according to the standard oven-drying method [30]. Accurately weighed samples (approximately 0.5 g) were placed in pre-dried and tarred ceramic dishes and dried in a convection oven at 100 ± 2 °C until a constant weight was achieved (typically for 3–4 h). The samples were cooled to room temperature in a desiccator before reweighing. The moisture content (%) was calculated as the percentage of weight loss relative to the initial sample weight. All measurements were performed in triplicate.

### 2.4. Bulk Density Measurement

The bulk density of native and irradiated β-glucan powders was determined using the graduated cylinder method [31]. Approximately 1 mL of the powder sample was gently poured into a pre-weighed 10 mL graduated glass cylinder without any tapping or compaction. The exact volume was adjusted to the 1 mL mark, and the cylinder with its content was weighed. The bulk density was calculated as the ratio of the sample weight (g) to its volume (mL), with the resulting value multiplied by 1000 to convert to kg/m^3^, according to pharmacopoeia guidelines. All measurements were performed in triplicate.

### 2.5. Solubility in Water

To determine the solubility, the powders of water-soluble glucan-containing preparations were first lyophilized. Then, 2 g of the powder sample were placed into a 50 mL glass centrifuge tube with a cap, mixed with 10 mL of deionized water on a vortex mixer for 20 min, and centrifuged at 6000 g for 20 min using a high-speed centrifuge CN-350 (HsiangTai Precision Co., Ltd., New Taipei City, Taiwan). The supernatant was separated, dried under vacuum at 60 °C for 2 h using a vacuum dryer, and weighed. The solubility (in mg/mL) was calculated using Equation (1):


(1) $$Solubility=\frac{Weight\;of\;dried\;supernatant}{Volume\;of\;supernatant}$$

### 2.6. Intrinsic Viscosity Determination

The viscosity of the solutions was determined by capillary viscometry using a VPZH-2 glass capillary viscometer (EKROSKHIM LCC, St. Petersburg, Russia) immersed in a water bath thermostatted at 30 °C. The glucan concentrations were selected in the range in which the relative viscosity was maintained between 1.2 and 2.0 to avoid the end effect. The relative viscosity of the solutions was determined using Equation (2):

(2) $$\eta_{rel}=\frac{\tau }{\tau_{0}}$$

where *τ* is the solution flow time, measured in s, and *τ*_0_ is the solvent flow time, also measured in s.

The characteristic viscosity [*η*] was determined using the Solomon-Ciutǎ mathematical models [32] according to Equation (3) and was calculated as the average value for three concentrations of glucan:

(3) $$\left[\eta \right]=\frac{\sqrt{2\cdot \left[\eta_{sp}-\ln\eta_{rel}\right]}}{c}$$

where *c* is the concentration of glucans, g/dL; *η_sp_* is the specific viscosity, defined as *η_rel_* − 1.

### 2.7. Congo Red Assay

To further confirm the glucans structure, a reaction with Congo red (CR) dye was used. This dye has high specificity for β-1,3-glucan chains. CR solution was prepared at a concentration of 20 μg/mL in physiological saline (0.9% NaCl). Native and irradiated β-glucan samples (25, 50, and 75 kGy) were dissolved in physiological saline to a final concentration of 400 μg/mL and alkalized with 0.1 M NaOH to pH~12.0 to induce the triple-helical conformation [33,34]. Equal volumes of alkalized β-glucan and CR solutions were mixed. After 10 min incubation at room temperature, absorption spectra were recorded in the range of 300–700 nm using UV-Vis spectroscopy on an SF-2000 spectrophotometer (OKB Spectr LLC, St. Petersburg, Russia). Free CR (20 μg/mL) in alkaline saline served as a control. The bathochromic shift (Δλ) was calculated as the difference in absorption maxima (λ_max_) between the CR-β-glucan complex and free CR.

### 2.8. DPPH Radical Scavenging Activity Assay

The antiradical activity of glucan-containing samples against the stable free radical 2,2-diphenyl-1-picryl-hydrazyl (DPPH, extra pure, 95%, Sisco, Mumbai, India) was studied using UV-Vis spectroscopy on an SF-2000 spectrophotometer (OKB Spectr LLC, St. Petersburg, Russia). A detailed description of the experiment was presented previously [35]. The concentration of glucan-containing samples was 2 mg/mL. Antiradical activity (ARA) was calculated using Equation (4):

(4) $$ARA=\frac{\left(A_{0}-A_{1}\right)}{A_{0}}\cdot 100\%$$

where *A*_0_ is the absorbance of the DPPH solution with water (blank); *A*_1_ is the absorbance of the DPPH solution with a solution of glucan-containing samples.

### 2.9. Dynamic Light Scattering (DLS) and Zeta Potential Measurement

The particle size of glucan-containing preparations in aqueous solution was determined by dynamic light scattering at 25 °C using a Photocor Compact-Z analyzer (Photocor LLC, Moscow, Russia) with a laser wavelength of 637.7 nm and a maximum light beam power of 25 mW at a scattering angle of 90°. Aqueous solutions of unirradiated and irradiated (75 kGy dose) glucan-containing preparations were prepared at a concentration of 100 μg/mL. Before measurements, the solutions were centrifuged. The particle size distribution was expressed as intensity and mass distributions obtained from the analysis of the correlation function. Data collection and analysis were performed using DynaLS software (Vers. 2.9.1, Dr. Alexander Goldin, Alango Ltd., Tirat Carmel, Israel). Zeta potential measurements using laser Doppler anemometry were performed on the same Photocor Compact-Z analyzer. Doppler shift analysis of the studied samples was performed using the PALS (Phase-Analysis Light Scattering) method at 25 °C. The stability of the particle size distribution and zeta potential was determined based on at least three measurements of each sample.

### 2.10. Collection of Honey Sample

The object of the study was polyfloral honey obtained from Carniolan honeybees (*Apis mellifera carnica*) during the period from April to June 2024 at the apiary of the Manurikov private subsidiary farm (Novgorod region, Russia). The study was conducted on three groups of bees, formed according to the principle of analogue families considering their weight, age, amount of brood and queen cells, drawn frames, and colony strength (one control group and two experimental groups, totaling 9 hives) during the main nectar flow. The experimental groups received 60% sugar syrup with the addition of a water-soluble fraction of irradiated and unirradiated glucan (0.01%) with a feeding frequency of once every three days, 200 g per bee colony. The bees were fed during the period of active vegetation and honey production (from 1 May to 1 September).

Hives № 1, 2, and 3 contained the control group bees; hives № 4, 5, and 6 contained bees receiving the water-soluble non-irradiated glucan-containing preparation; and hives № 7, 8, and 9 contained bees receiving the water-soluble irradiated glucan-containing preparation. The control group received sugar syrup without additives. The honey was collected in 2024. Although the honey was collected and extracted in 2024, the study continued until 2026. This was due to the study’s aim to study the stability of honey quality during long-term storage (up to 24 months). The honey was stored in a refrigerator at 8–10 °C.

### 2.11. Proline Content Determination

The mass fraction of proline in honey was determined by dissolving honey in water, allowing proline to react with ninhydrin to form a colored complex, measuring the absorbance of the test solution and the reference solution, and then calculating the proline content X (mg/kg) [36]. This standard applies to honey and establishes a method for determining the mass fraction of proline in the measurement range from 170.00 to 770.00 mg/kg. An aqueous solution of L-proline (99.0%.) with a concentration of 32 μg/mL was used as a standard solution. Ninhydrin monohydrate was dissolved in ethylene glycol monomethyl ether at a concentration of 30.0 mg/mL. Honey samples weighing at least 200 g were collected for analysis. Comb honey (without sill cells) was separated from the comb using a sieve without heating. Crystallized honey (for analysis in 2026) was softened in a thermostatically controlled water bath at a temperature no higher than 40 °C. The sample was cooled to room temperature. A 5 g sample of prepared honey was weighed into a 50 mL beaker, 10 mL of distilled water was added, the honey was thoroughly ground with a glass rod, and the liquid was transferred to a 100 mL measuring flask. This process was repeated two or three times until the honey was completely dissolved. The beaker was then rinsed several times with small portions of water, which were also poured into the measuring flask, ensuring that the volume of liquid did not exceed two-thirds of the flask’s volume. The solution in the flask was brought to the mark with water and thoroughly mixed. Three 25 mL test tubes were filled with 0.5 mL of honey solution (test solution), 0.5 mL of distilled water (blank), and 0.5 mL of standard L-proline solution. To each tube was added 1 mL of ninhydrin solution and 1 mL of formic acid. The contents of the tubes were thoroughly mixed after each addition. The tubes were tightly capped and placed in a constantly boiling water bath, ensuring that the water level in the bath was above the liquid level in the tubes. The tubes with the liquids were kept in the boiling water bath for 15 min, then transferred to a water bath heated to 70 °C. A total of 5 mL of 50% isopropyl alcohol was added to each tube and quickly capped again. The test tubes containing the liquids were kept in a water bath for 10 min, then cooled at room temperature for 45 min. Using an SF-2000 spectrophotometer (OKB Spectr LLC, St. Petersburg, Russia), the maximum absorption of the test (*A_p_*) and standard (*A_s_*) solutions was measured relative to the control sample at a wavelength of 510 nm.

The test results were taken as the arithmetic mean of six parallel determinations of proline content obtained under repeatability conditions, provided that the absolute discrepancy between parallel determinations did not exceed the repeatability limit of 0.073$\bar{X}$.

The proline content is calculated using Equation (5):

(5) $$X=80\cdot {\bar{A}}_{p}\cdot m_{1}\cdot {\bar{A}}_{s}^{-1}\cdot m_{2}^{-1}$$

where 80 is the dilution factor per 1 kg of honey; ${\overset{¯}{A}}_{p}$ is the arithmetic mean absorbance of the test solution; $m_{1}=40$ is the mass of proline in the standard solution (mg); ${\overset{¯}{A}}_{s}$ is the arithmetic mean absorbance of the proline standard solution; $m_{2}=5.0$ is the mass of the analyzed honey sample (g).

### 2.12. Statistical Analysis

To identify significant differences in proline content in honey from different bee groups, data were analyzed using a *t*-test for two independent sample means using Origin software (Version 9.2, Origin Lab Corporation, Northampton, MA, USA). Differences between groups were considered significant at a significance level of less than 0.05. Data are presented as mean ± standard deviation.

## 3. Results and Discussion

### 3.1. Physicochemical Characterization of Glucan-Containing Water-Soluble Preparations Isolated from P. Ostreatus

**Figure 1:**
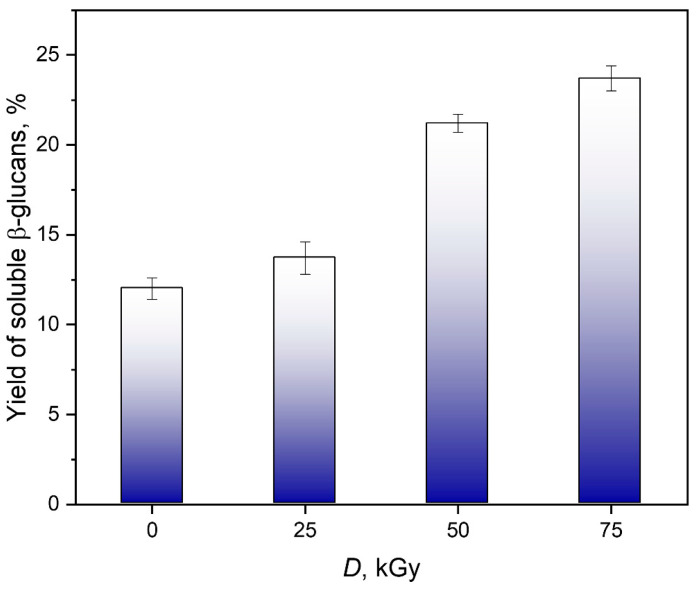
Dependence of the yield of water-soluble glucans on the absorbed dose.

Extraction of glucans with hot water is a simple and easily implemented method, but it has some disadvantages such as a relatively long reaction time, high temperature, high energy consumption, and relatively low extraction efficiency [37]. Despite the fact that the effect of ionizing radiation at different stages of glucan extraction is used primarily for their depolymerization and, accordingly, for obtaining low-molecular products, this treatment can also lead to an increase in the yield of water-soluble glucan fractions [38]. An analysis of the dependence of the yield of water-soluble glucan-containing preparations obtained in our study on the absorbed dose of ionizing radiation also revealed the presence of a direct positive correlation: with an increase in the dose, an increase in the yield of the water-soluble product is observed (Figure 1).

Table 2 presents the results of a study of the physicochemical characteristics of water-soluble glucan-containing preparations depending on the absorbed dose of ionizing radiation. Analysis of the obtained data showed that an increase in the absorbed dose affects both the appearance and a number of physicochemical parameters of the studied samples. For example, irradiation led to a change in the color of the preparation from light brown to light yellow. This color change is considered a preliminary macroscopic indicator of physicochemical changes caused by exposure to ionizing radiation, likely associated with limited oxidative changes and structural rearrangement of β-glucan [39].

Water solubility, bulk density, and moisture content in samples exposed to electron irradiation changes dose-dependently. An increase in the yield of the water-soluble fraction in irradiated β-glucans, along with an increase in solubility, was also observed upon irradiation of (1→3)-β-D-glucans isolated from the cell wall of brewer’s yeast [38]. However, in our study, the water-soluble content and solubility of the glucan samples did not increase linearly with increasing radiation dose. The relatively small increase in solubility is likely due to partial depolymerization of β-glucan chains during irradiation, resulting in the formation of lower molecular weight fragments with improved hydration properties [39].

β-glucans possess high moisture content and the ability to retain water, primarily due to their highly hydrophilic molecular structure. We observed a dose-dependent decrease in the moisture content of our samples, which we attribute to both partial radiation-induced polymer degradation, accompanied by the disruption of interchain hydrogen bonds and, consequently, the partial release of bound water; and the destruction of bound water molecules due to direct radiolysis. These considerations also logically correlate with the dose-dependent decrease in bulk density: an increase in porosity due to water radiolysis leads to a decrease in the overall mass of the sample, and shorter polymer chains formed as a result of radiation degradation are less densely packed. A decrease in the bulk density of tapioca starch was also observed upon exposure to gamma radiation [40].

As can be seen from Table 2, compared to the unirradiated sample, an increase in intrinsic viscosity was observed with irradiation at a dose of 25 kGy. It then decreased to the values of the unirradiated sample as the dose increased to 75 kGy. We hypothesize that the initial increase in viscosity at the lowest dose studied is due to radiation-induced destruction of high-molecular-weight β-glucans, previously insoluble in water, and their transfer to the aqueous extract. Thus, an increase in the proportion of β-glucans should be expected in the sample irradiated at a dose of 25 kGy. It should be noted that the intrinsic viscosity value we obtained for the unirradiated sample is in good agreement with the molecular weight of β-glucan-containing preparations (*M_w_* = 33 kDa) extracted from *P. ostreatus* biomass using a similar method [41]. With increasing doses from 25 to 75 kGy, the observed decrease in intrinsic viscosity is associated with the destruction of higher molecular weight β-glucans additionally extracted from the insoluble sample. Our hypothesized processes are consistent with the increase in the yield of the water-soluble fraction with increasing dose, indicating radiation-induced degradation of some previously insoluble glucans and their conversion to a water-soluble fraction due to a decrease in molecular weight. It is also worth noting that in the literature reviewed, radiation-induced degradation of β-glucans was performed at other stages. Typically, the finished water-soluble β-glucans was irradiated in solution [9,18,19,42] or powder [14,39,43], and a dose-dependent decrease in viscosity and molecular weight was observed, consistent with our results at doses of 25–75 kGy.

Thus, irradiation of samples of β-glucan-containing preparations contributed to an increase in solubility in water and a decrease in bulk density and moisture content of the released water-soluble fraction, which may be associated with changes in the structure of the material under the influence of ionizing radiation at the micro- and macro-level.

### 3.2. Effect of Irradiation on Particle Size Distribution and Zeta Potential

**Figure 2:**
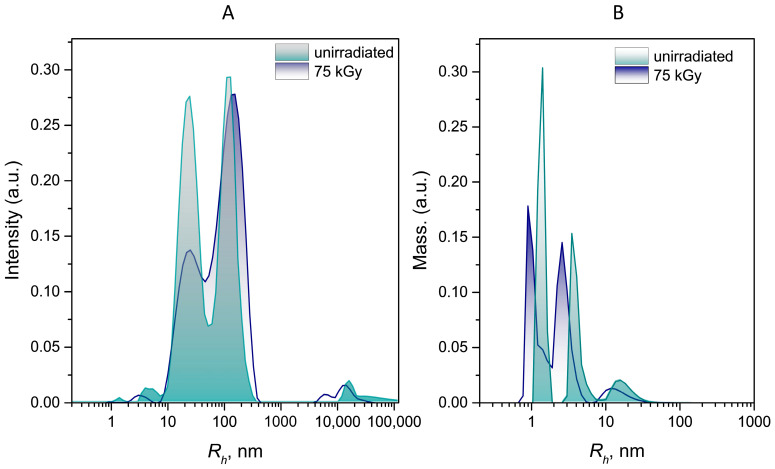
Particle size distributions of unirradiated and irradiated glucan-containing samples weighted by light scattering intensity (**A**) and by mass (**B**).

The potential use of water-soluble β-glucans for biomedical applications requires the evaluation of particle size in aqueous solutions and their zeta potential. Figure 2 shows the particle size distributions of unirradiated β-glucans and those subjected to electron irradiation at a dose of 75 kGy. Since large particles and aggregates contribute the most to light scattering, the scattering intensity-weighted particle size distribution (Figure 2A) was converted to a mass distribution (Figure 2B) using model dependences of light scattering intensity on particle size. The key observation is that both irradiated and unirradiated particles exhibit a trimodal distribution. The hydrodynamic radius of about 4 nm of the average mode in the particle mass distribution observed for the unirradiated polysaccharide is in good agreement with the data obtained on the low molecular weight (*M_w_* = 33 kDa) of water-soluble glucans isolated from the fruiting bodies of *P. ostreatus* by hot water extraction and subsequent ethanol precipitation [41], as was done in our study. It can be assumed that particles with a size of approximately 1.5 nm, observed in unirradiated solutions of the glucan-containing preparation, belong to proteins, the presence of which is confirmed below by the solid-state ^13^C NMR spectroscopy and is also consistent with the data presented by Xia et al. [41], indicating that the water-soluble fraction obtained from *P. ostreatus* by hot-water extraction contains a significant amount of protein (24.4 wt. %). Particles with a size of approximately 11 nm may correspond to aggregated particles of β-glucans, the large number of hydroxyl groups in the structure of which facilitates their aggregation through hydrogen bonds. As can be seen, the particle distribution of glucan-containing samples irradiated with a dose of 75 kGy shifts toward smaller sizes. This phenomenon is in good agreement with numerous published data confirming the radiation degradation of β-glucans of various natural origins, irradiated both in solution and in powder [38,39,42,43,44]. Furthermore, it should be noted that glucan-containing fractions, even isolated from a single natural source—oyster mushrooms—but using different extraction methods, differ significantly in both composition and molecular weight, and, accordingly, in the behavior of polysaccharide particles in solutions [45,46].

Another characteristic that determines the stability of a particle suspension is the zeta-potential. Particles with a high absolute zeta potential (more than 30 mV) are less prone to aggregation due to electrostatic repulsion between them. Initially, β-glucans, which are non-ionic polysaccharides, have a zeta-potential close to neutral [47]. According to our data, the unirradiated glucan-containing preparation had a zeta potential of −2.2 ± 0.7 mV, which increased negatively to −5.4 ± 1.1 mV in the sample irradiated with a dose of 75 kGy. Thus, irradiation contributed insignificantly to the increase in the stability of aggregation of preparations containing glucan. The obtained data are consistent with the presented data indicating that irradiation leads to a moderate increase in surface charge [48].

### 3.3. Structural Characterization

The composition, structure, and conformation of the obtained water-soluble β-glucan-containing products in solution were determined using FTIR, NMR and UV-Vis-spectroscopy using Congo Red dye.

#### 3.3.1. FTIR Spectroscopy

**Figure 3:**
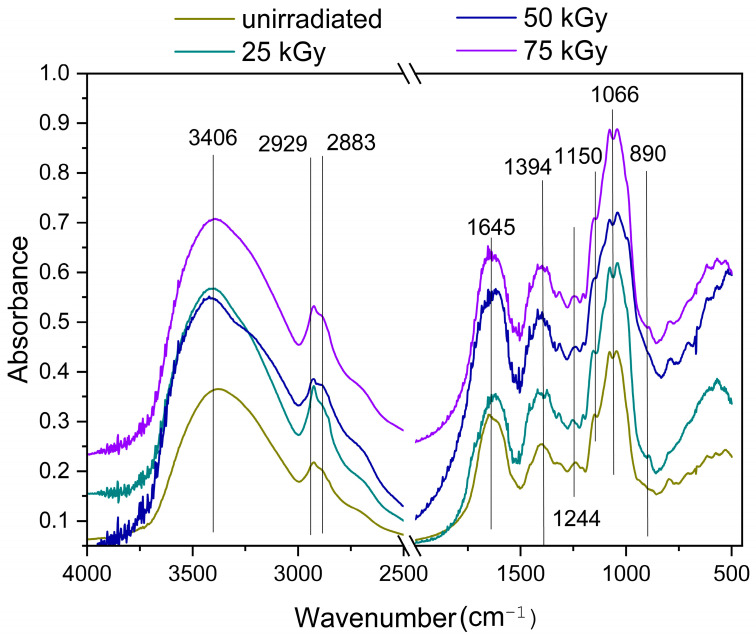
FTIR spectra of unirradiated and irradiated glucan-containing water-soluble samples at 25–75 kGy.

Figure 3 shows the FTIR spectra of water-soluble glucan-containing samples extracted from unirradiated and irradiated alcohol-treated cryopowder from *P. ostreatus* biomass. Both irradiated and unirradiated samples exhibited a characteristic absorption peak at 890 cm^−1^, indicating the presence of β-(1→3) and β-(1→6) glycosidic bonds (pyranose ring vibrations) [49,50,51]. The intense band at 1069 cm^−1^ corresponds to the stretching vibrations of the C-C bonds in the pyranose ring and the C-O stretching vibrations of the glycosidic bond C−OH [52]. The peaks at 1645 cm^−1^ and 3406 cm^−1^ are due to the deformation vibrations of bound water [9] and the stretching vibrations of the –OH group, respectively. The shoulder in the region of 3100 cm^−1^ can be attributed to vibrations of the N–H bond in chitin, and the bands at 2930–2880 cm^−1^ to asymmetric/symmetric stretching of the C–H bond of the polysaccharide chains [53]. In the regions of 1630 cm^−1^ and 1420–1380 cm^−1^, absorption bands corresponding to polysaccharides were observed [54]. A band at 1150 cm^−1^, representing vibrations of bonds in α-linked glucans, is also observed in all samples [55]. The bands characteristic of α-glucan were detected. The IR bands characteristic of α-chitin at 1650–1630 cm^−1^, 1560 cm^−1^, 1382 cm^−1^ and 1318 cm^−1^ are of weak intensity, indicating a low chitin content in the samples, which is consistent with data obtained previously for glucans extracted from *P. ostreatus* [46]. Figure 3 also shows that the characteristic absorption peaks and peak positions of the water-soluble glucan-containing samples obtained from both the irradiated and unirradiated samples do not differ significantly, indicating that no new functional groups were formed during the irradiation process, and the main functional groups of the β-glucan chain still exist, indicating that the β-glucan structure was not destroyed or altered. However, the intensity of β-glucan peaks in irradiated samples is higher than in the non-irradiated sample, which may be due to a decrease in the number of intramolecular hydrogen bonds and greater accessibility of functional groups due to radiation-induced destruction of the polysaccharide chain.

#### 3.3.2. Nuclear Magnetic Resonance Spectroscopy

**Figure 4:**
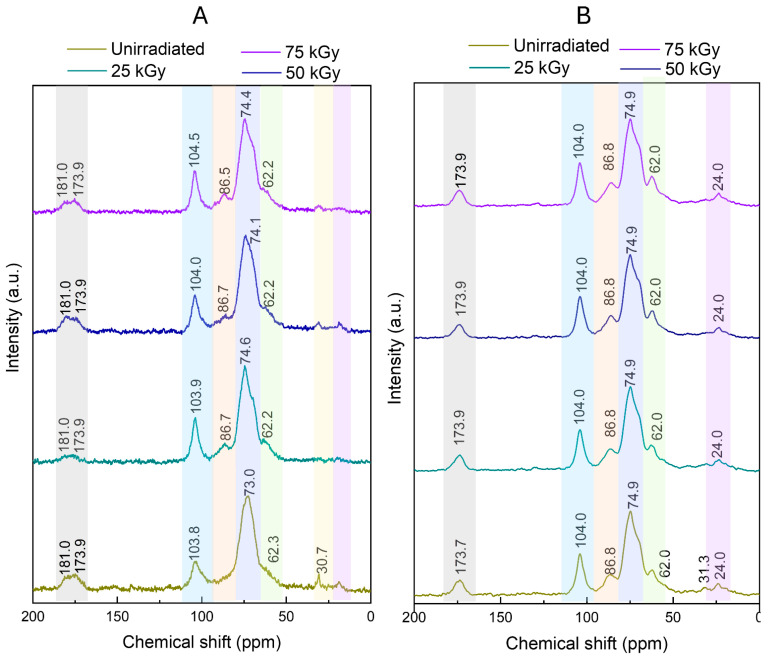
A solid-state ^13^C CP/MAS NMR spectra for water-soluble (**A**) and insoluble (**B**) β-glucans isolated from *P. ostreatus*.

**Figure 5:**
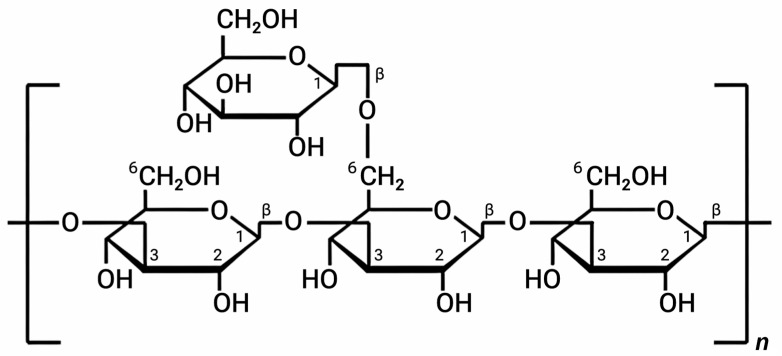
Chemical structure of branched β-(1→3,1→6)-D-glucans.

Solid-state ^13^C CP/MAS NMR spectroscopy was used as a complementary method to confirm the structure of the isolated samples and to investigate the effect of ionizing radiation on the purity and structural properties of the β-D-glucan-containing preparation isolated from *P. ostreatus*. The bands with chemical shifts at 104, 75.9, 85, 70.9, 77.1, and 68.7 ppm present in the spectra of water-soluble samples (Figure 4) correspond to C1, C2, C3, C4, C5, and C6 of branched β-(1→3,1→6)-D-glucans [53,56,57,58,59,60,61,62,63], the chemical structures of which are shown in Figure 5.

It should be noted that the interpretation of the part of the ^13^C CP/MAS ss NMR spectrum in the range of 55–85 ppm is quite complex, since in this range of chemical shifts, in addition to the signals of C5, C2, and C4 (1→6)-linked carbon atoms and the signals of C3 and C6 β-(1→3,1→6)-D-glucan, signals of C2, C3, C4, C5, and C6 β-(1→3)-glucans and β-(1→4)-glucans can be observed [56].

The ^13^C CP/MAS NMR spectra of all insoluble fraction samples (Figure 4B) show fairly intense peaks at 174 and 24 ppm, indicating the presence of chitin (carbonyl and methyl groups of chitin, respectively) [64]. The main difference in the ^13^C CP/MAS NMR spectra of both irradiated and unirradiated water-soluble samples (Figure 4A) is the presence of peaks in the region of 181 ppm. These maxima are a characteristic feature of ^13^C NMR spectra of β-glucan-containing fractions isolated from *P. ostreatus* [9] due to protein contamination [41]. In addition, the presence of proteins is confirmed by a broad peak of the carbonyl group (about 175 ppm) and peaks of aliphatic carbon atoms (about 30 ppm) [65]. According to other data, the shift of the carbonyl group signal up to 180 ppm in all water-soluble fractions compared to the insoluble fraction may be due to chemical modification, structural changes or degradation of chitin [56]. Since chitin’s solubility depends significantly on its molecular weight, it can be assumed that low-molecular-weight chitin derivatives are present in the soluble fraction. Although the yield of radiation-induced chitin degradation is quite low, its molecular weight decreases under irradiation, and it undergoes deacetylation, resulting in the formation of free primary amino groups. Radiation-induced degradation results in the formation of compounds with carboxyl groups [66]. The presence of a small amount of α-linked glucans in the samples, obtained using FTIR spectroscopy, is confirmed by the presence of a weakly expressed shoulder in the region of 101 ppm, corresponding to the signal C1 of α-(1→3)-glucan [62,64].

**Figure 6:**
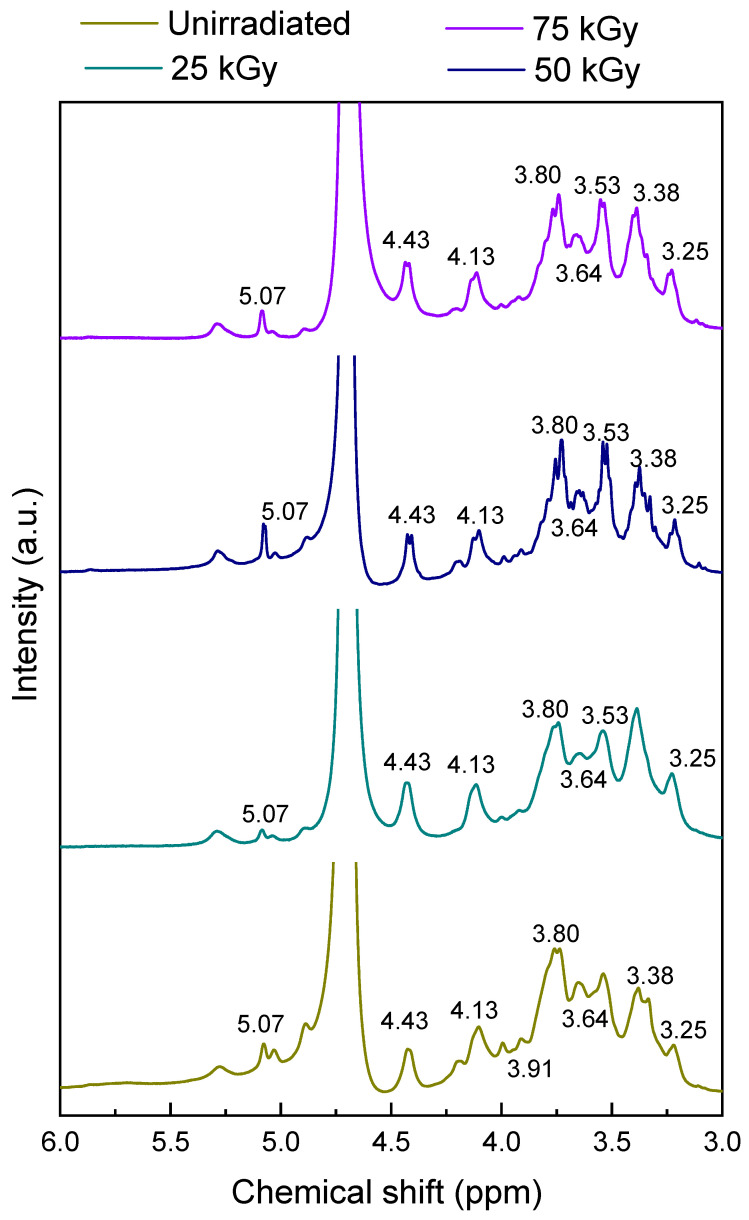
^1^H NMR spectra of polysaccharides isolated from water-soluble fractions of fruiting bodies of *P. ostreatus*, recorded at 400 Hz in D_2_O at room temperature.

Liquid ^1^H NMR spectroscopy was used to further characterize the structure of polysaccharides from *P. ostreatus* (Figure 6). In the ^1^H NMR spectrum, the α- or β-configuration of the glycosidic bond is mainly determined by the proton peak at the anomeric C1. For glucans, the chemical shift of H1 in the range of 4.8–5.3 ppm corresponds to α-glycosidic bonds, while H1 of β-glycosides appears at 4.4–4.8 ppm [53,67,68]. The spectra of all water-soluble samples (Figure 6) showed the same pattern characteristic of β-(1→3,1→6)-D-glucans [43,63]. Thus, the signals in the region of 4.43 ppm, 3.25 ppm, 4.13 ppm, 3.38 ppm, 3.53 ppm correspond to the anomeric protons H1, protons H2, H3, H4, H5 of β-linked glucopyranosyl units [53,59,63,69]. Due to the chiral structure of glucose, the C6 atom contains two diastereotopic hydrogen atoms H6a and H6b [70], which appear in ^1^H NMR spectra in two places, namely in the region of about 3.7–3.8 and 3.5–3.6 ppm.

Thus, according to FTIR and NMR spectroscopy data, water-soluble samples isolated from both irradiated and unirradiated preparations are β-(1→3,1→6)-D-glucans contaminated with proteins and a small amount of (1→3)-α-glucan.

### 3.4. Effect of Irradiation on the Conformation of β-(1→3,1→6)-D-Glucans

**Figure 7:**
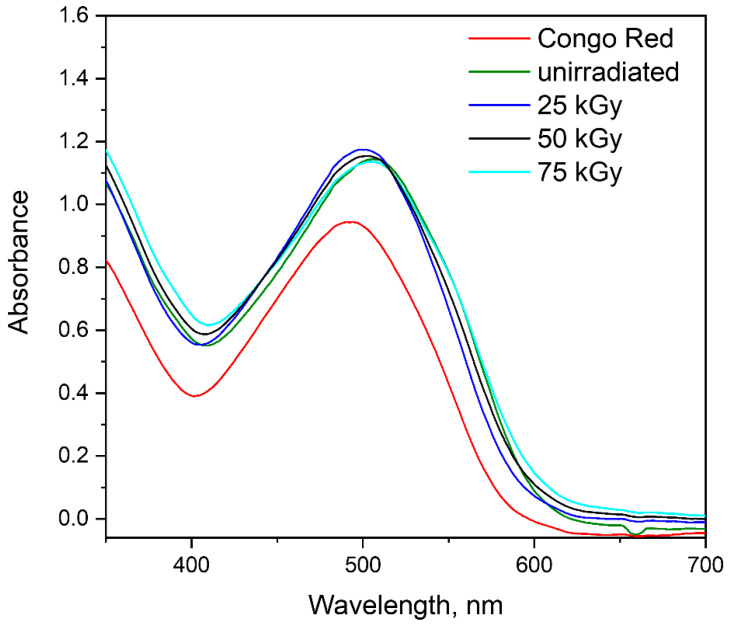
Absorption spectra of the Congo red- β-(1→3,1→6)-D-glucan complexes.

The triple-helical conformation of β-glucans is not merely a structural feature but a prerequisite for their biological recognition by immune receptors, including Dectin-1 and complement receptor CR3. This ordered architecture enables multivalent interactions with receptor clusters, triggering downstream signaling cascades and subsequent immunomodulatory responses [71,72]. Therefore, any structural alteration induced by processing—such as γ- or E-beam irradiation—could potentially compromise bioactivity. In this context, evaluating the conformational integrity of irradiated β-glucan samples is of critical importance. The Congo Red (CR) binding assay, originally developed for the detection of ordered triple-helical conformations in β-glucans, was employed to assess the effect of E-beam irradiation on the higher-order structure of the polysaccharide. In dilute alkaline media (0.1–0.15 M NaOH), native β-glucan adopts an ordered triple-helical conformation stabilized by a cooperative network of inter- and intramolecular hydrogen bonds. The linear anionic diazo dye CR exhibits high binding affinity for the hydrophobic pockets and helical grooves of this supramolecular structure. The resulting complex formation is accompanied by a characteristic bathochromic shift of the CR absorption maximum from approximately 490 nm to 505–530 nm, which is widely employed as a diagnostic feature of triple-helical integrity [63,73,74]. The CR with native and E-beam-irradiated β-glucan samples (25, 50, and 75 kGy) was studied to assess the integrity of the triple-helical conformation. Free CR exhibited an absorption maximum at 488 nm. Upon complexation with β-glucan, all tested samples induced a significant bathochromic shift to the range of 508–516 nm (Figure 7). The magnitude of the shift (Δλ) varied between 20 and 28 nm across all samples, with no statistically significant differences observed between the unirradiated and irradiated groups. These results provide clear evidence that the triple-helical conformation of β-glucan remains intact after E-beam irradiation at doses up to 75 kGy. Although radiation may cause localized chain scissions, the overall helical architecture is preserved due to the cooperative network of inter- and intramolecular hydrogen bonds stabilizing the triple helix. This observation is in good agreement with previous studies demonstrating the high conformational stability of β-glucans under various processing conditions [42,44,75]. Thus, we assume that soluble β-glucans extracted from an insoluble glucan-containing preparation irradiated with an electron beam at doses of up to 75 kGy retain their conformation in solution, which is important from the point of view of preserving the functional properties of the obtained products.

### 3.5. Evaluation of Antiradical Activity of β-(1→3,1→6)-D-Glucans

**Figure 8:**
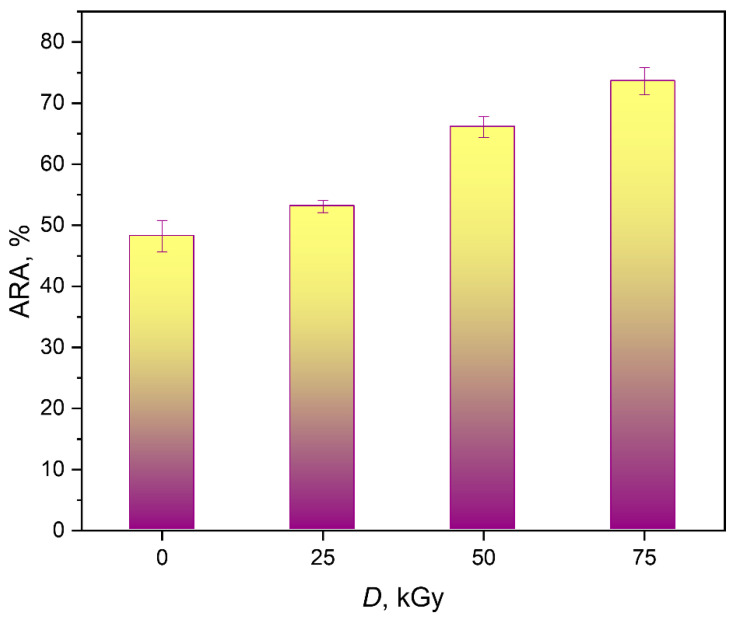
Results of the evaluation of the antiradical activity of β-(1→3,1→6)-D-glucans with the stable free radical DPPH.

Free radicals are constantly generated in the body during metabolism, and they are maintained in balance with antioxidants. When this balance is disrupted, large amounts of reactive oxygen species (ROS) are produced. Excessive amounts of these can attack cells, damaging them and causing various diseases, as well as deteriorating overall health. Since β-glucans are known to have antioxidant properties, we assessed whether exposure to ionizing radiation affects this parameter. As Figure 8 shows, irradiation leads to a dose-dependent increase in the antioxidant activity of β-glucans. The greatest effect was observed at a dose of 75 kGy, where the ARA value increased from 48.2% to 73.6%.

**Figure 9:**
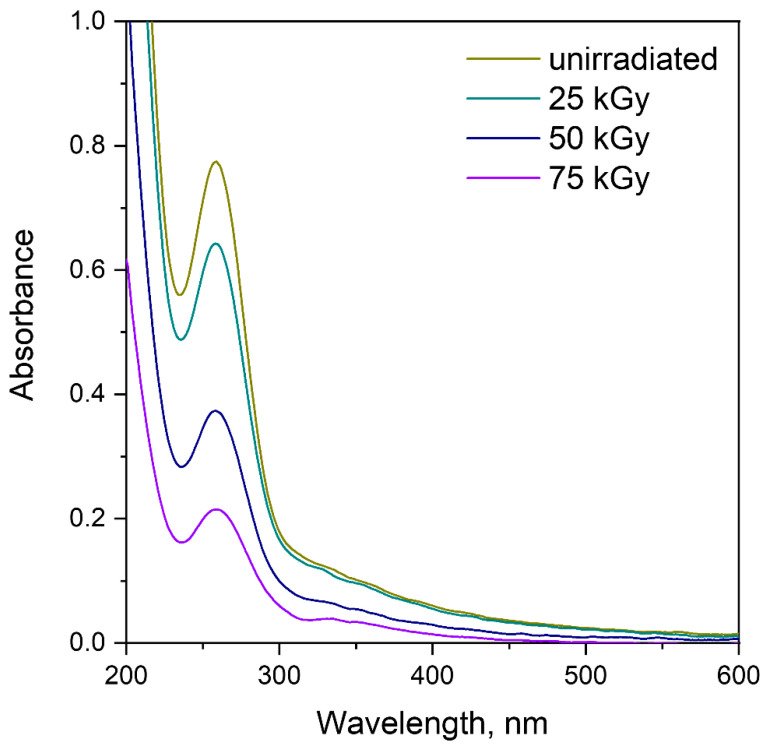
UV-Vis spectra of unirradiated and irradiated at 25–75 kGy of β-(1→3,1→6)-D-glucan solutions.

Similar effects of increasing antiradical activity were also obtained with gamma irradiation of β-glucan-containing preparations isolated from both *P. ostreatus* [41], and other natural sources [49]. In some studies, the authors suggest that the increase in antiradical activity is associated with the formation of new double bonds in the polysaccharide [14]. However, in our study this assumption is not supported by NMR and FTIR spectroscopy data. In addition, as can be seen from the UV-Vis spectra presented in Figure 9, the absorption of irradiated β-glucans solutions decreased dose-dependently, which further confirms that in our case the antiradical activity increases for other reasons. Possible reasons for the increase in β-glucan activity under the influence of radiation may include partial depolymerization, accompanied by rupture of the glycosidic chain and a decrease in the number of intramolecular hydrogen bonds, leading to the release of functional hydroxyl groups, the protons of which are capable of reducing DPPH, as well as contamination with proteins and artifacts specific to this analysis.

### 3.6. The Effect of Adding β-(1→3,1→6)-D-Glucans to the Diet of Bees on the Proline Content of Honey

The development of a honeybee diet enriched with natural additives of natural origin that can stimulate the immune system is a promising area of research, which has been confirmed by the use of β-(1→3,1→6)-D-glucans as a food additive. As has been shown previously, the introduction of 0.5% (*w*/*w*) β-(1→3,1→6)-D -glucans into the diet not only had no effect on the survival of newborn and nursing bees, but also stimulated the activation of phenoloxidase activity in honey-fed bees [24], which confirms the ability of β-glucans to influence the immune system of honey bees. β-(1→3,1→6)-D -glucans as a dietary supplement were shown to reduce the viral load in bees infected with deformed wing virus and promote higher survival compared to control groups [76]. Proline, the most abundant free amino acid in honey [77] is a critical indicator of honey quality, determining its maturity, naturalness, and the possibility of adulteration. Minimum standards for this amino acid content are often set at 180–300 mg/kg. Proline performs multiple functions: it acts as an osmoprotectant, antioxidant, and energy reserve for immune cells. It is known that proline rapidly accumulates in the hemolymph of insects during immune stress [26]; therefore, the addition of immunomodulatory substances to the diet of bees should result in an increase in the content of this amino acid in honey. We investigated the effect of beta-glucan on proline content in honey, since it is, on the one hand, an important biomarker of bee health [26,27,28], and at the same time its content is a recognized standardized indicator of honey authenticity and quality [29].

**Figure 10:**
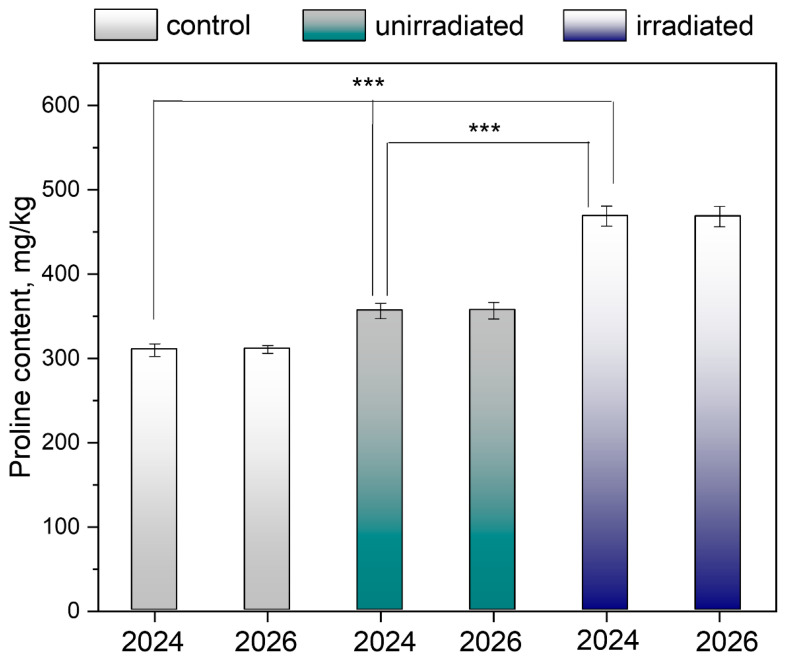
Proline content in polyfloral honey of Carniolan honey bees (*Apis mellifera carnica*) *** The difference between the two groups is statistically significant (*p* < 0.001).

In this study, Carniolan honeybees (*Apis mellifera carnica*) were fed a dietary supplement of irradiated and unirradiated glucans. As can be seen in Figure 10, the addition of both irradiated and unirradiated β-glucans statistically significantly (*p* < 0.001) increased the proline content in honey. Proline content was highest in bees fed radiation-treated glucan, and the difference between proline content in honey from bees fed unirradiated and irradiated β-glucan was also statistically significant (*p* < 0.001). Importantly, proline content, assessed after two years of honey storage at 8–10 °C, decreased only slightly.

Thus, the obtained data indicate that radiation treatment of β-glucan not only contributed to the preservation of their biological activity, but also significantly increased it.

## 4. Conclusions

This study assessed the effects of electron beam irradiation of β-glucans extracted from cryopowder obtained from *P. ostreatus* fruiting bodies in the 25–75 kGy range. Radiation treatment was found to increase the yield of water-soluble glucan-containing products and enhance their solubility in water. Structural analysis revealed that water-soluble glucan samples isolated from both irradiated and unirradiated samples were β-(1→3,1→6)-D-glucans contaminated with proteins and a small amount of (1→3)-α-glucan. Insoluble products of hot-water extraction contained chitin in addition to the aforementioned fractions. Radiation treatment did not significantly alter the functional groups or molecular organization, such that the primary polysaccharide backbone was largely preserved after irradiation. Particle size analysis revealed a decrease in size with a narrower distribution after irradiation, indicating fragmentation of β-glucan chains. However, even fragmented β-(1→3,1→6)-D-glucans retained a triple-helix conformation, which is important for preserving glucan receptor binding and immunomodulatory activity. Radiation treatment increased the antiradical activity of glucan-containing samples. Thus, radiation treatment can be used to produce more soluble (bioavailable) preparations, as evidenced by increased proline content in honey obtained from irradiated β-glucans consumed as a dietary supplement by Carniolan bees. Furthermore, sterilization can be achieved in parallel with the reduction in molecular weight of polysaccharides through radiation destruction. Despite the results obtained in this study, certain limitations of this work should be acknowledged. Our study focused primarily on the physicochemical and structural characterization of irradiated glucans, while the effect of irradiation on the rheological properties of glucans, their molecular weight distribution, and the relationship of these parameters with the biological activity of the studied biopolymer will be discussed further. However, the enormous potential of radiation-modified β-glucan is already evident: the identified ability to manipulate the metabolic “depot” of proline and its signaling functions opens new horizons for understanding how β-glucan regulates not only immune cells but also the balance between inflammation, tissue regeneration, and metabolic health in general.

## Figures and Tables

**Table 1:** The weights of water-soluble preparation.

Absorbed Dose, kGy	Weight, g
0	3.8 ± 0.2
25	4.4 ± 0.3
50	6.8 ± 0.2
75	7.6 ± 0.2

**Table 2:** Dependence of the physicochemical characteristics of a glucan-containing samples on the absorbed dose.

Absorbed Dose, kGy	Appearance	Solubility in Water, g/100 mL	Bulk Density, kg/m^3^	Moisture Content, %	Intrinsic Viscosity, dL/g
0	Light brown powder of uniform color	2.9 ± 0.1	362.1 ± 1.6	5.9 ± 0.5	0.76 ± 0.04
25	Light yellow powder of uniform color	3.0 ± 0.1	338.2 ± 1.2	3.3 ± 0.6	0.97 ± 0.06
50	Light yellow powder of uniform color	3.1 ± 0.1	325.6 ± 1.1	2.9 ± 0.2	0.84 ± 0.05
75	Light yellow powder of uniform color	3.5 ± 0.1	319.7 ± 1.4	2.7 ± 0.3	0.75 ± 0.01

## Data Availability

The original contributions presented in this study are included in the article. Further inquiries can be directed to the corresponding author.
